# Administration of Castor Oil

**Published:** 1873-12

**Authors:** E. Gregory


*AMER. JOURNAL OF PHARMACY—November and December,* 1873.

Administration of Castor Oil.—*By E. Gregory.*—Castor Oil is indisputably nauseous and unpleasant to take, so much so that some patients can not be induced to swallow it, by any device or on any consideration. At the same time its qualities are such that in some disturbed states of the system no other purgative can be substituted with safety.

For some twelve or fourteen years past I have used the following formula for a Castor Oil draught, which has proved very acceptable to adults who could not get down the pure oil. For children it does not answer so well, the dose of necessity being double that of the oil:

℞.—Ol. Ricini, . . . . . . . . ℥j.

Mucil. Acaciaæ, . . . . . . . ʒij.

Shake well together, then add—

Syr. Simp., . . . . . . . . ʒij.

Shake again, then flavor with Spts. Menthæ Pip., or according to taste, and make up two ounces with water. This mixture can scarcely be called an emulsion, but it mixes well on vigorous shaking. The taste is well disguised; it is thin enough to be easily taken from a wine glass, and it leaves no oil sticking around the mouth. I have lately obtained still better results from the following formula:

℞.—Ol. Ricini, . . . . . . . ℥ j.
Ol. Anisi, . . . . . . gtt x.
Chloroformi, . . . . . . gtt x.
Shake well together, then add—
Mucil. Acaciæ, . . . . . . . ℥ ss.
Shake well and make up to 2 ozs. with water.

